# Supplementary material for: Time-Resolved Visualization of Cyanotoxin Synthesis via Labeling by the Click Reaction in the Bloom-Forming Cyanobacteria Microcystis aeruginosa and Planktothrix agardhii
Source: Toxins (Basel). 2025 Jun 3;17(6):278. doi: 10.3390/toxins17060278 (PMC12197485; doi:10.3390/toxins17060278)
Supplement: Supplementary file 1 [file toxins-17-00278-s001.zip › toxins-3558256-supplementary.pdf]

Supplementary material: Time-Resolved Visualization of Cyanotoxin Synthesis via Labeling by the Click Reaction in the Bloom-Forming Cyanobacteria *Microcystis aeruginosa* and *Planktothrix agardhii*

Rainer Kurmayer and Rubén Morón Asensio

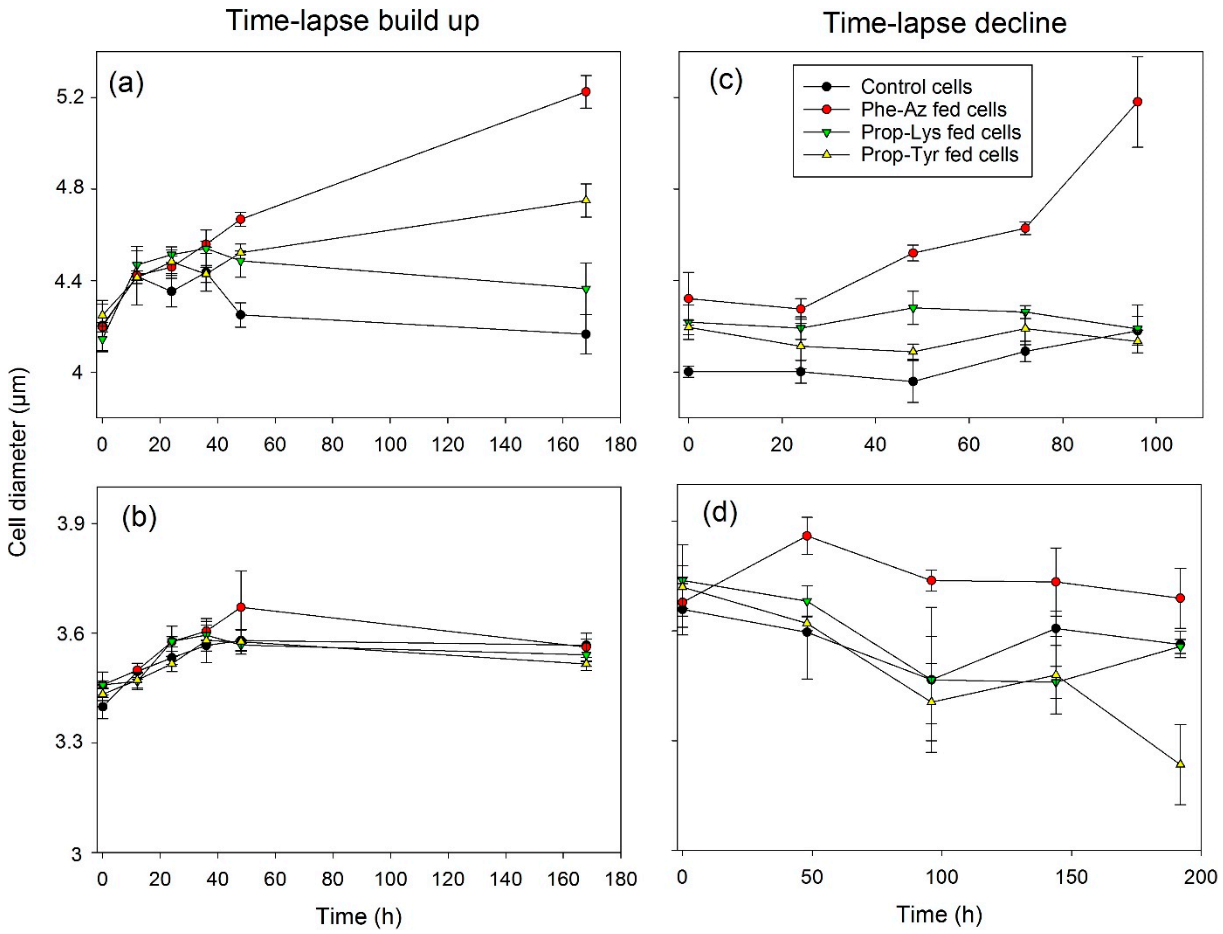

Figure S1: Mean (± SE) cell diameter of *M. aeruginosa* strain Hofbauer (a, c) or *P. agardhii* strain no371/1 (b, d) during time-lapse experiments related to the build up (a, b) or decline (c, d) of clickable MC (a, c) or clickable AP (b, d). Control cells were grown in the absence of non-AAs and processed under identical conditions.

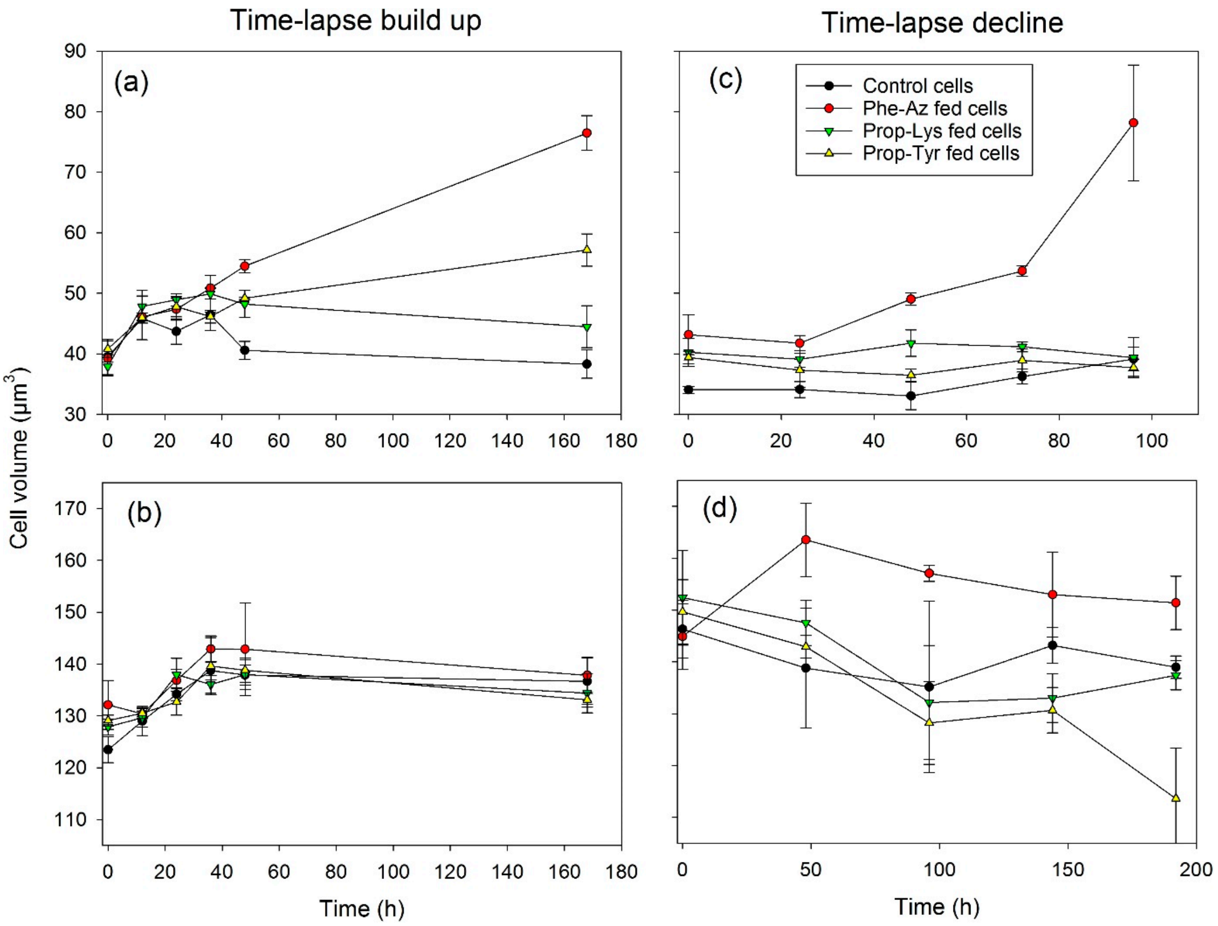

Figure S2: Mean (± SE) cell volume of *M. aeruginosa* (a, c) or *P. agardhii* (b, d) during time-lapse experiments related to the build up (a, b) or decline (c, d) of clickable MC (a, c) or AP (b, d).

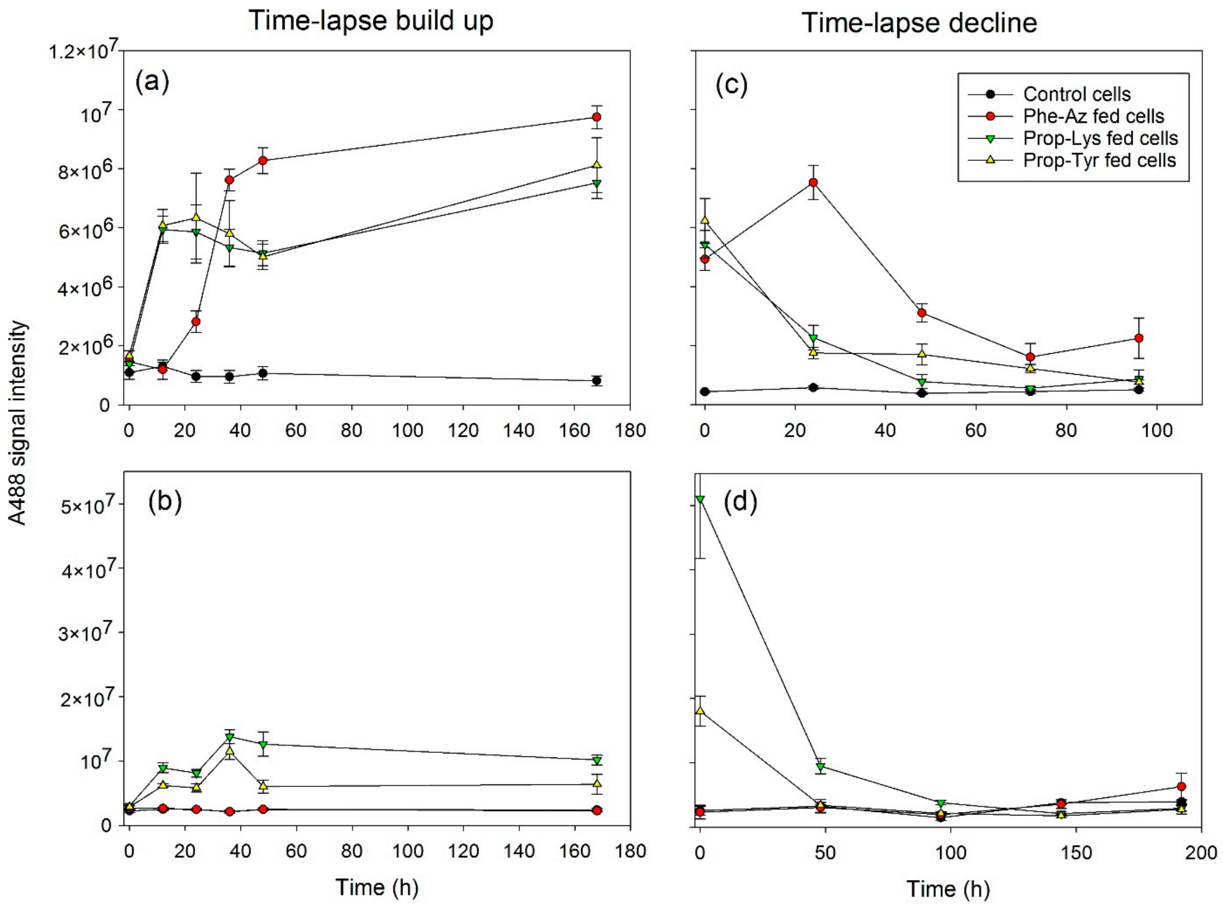

Figure S3: Mean (± SE) A488 signal intensity of *M. aeruginosa* (a, c) or *P. agardhii* (b, d) per cell during time-lapse experiments related to the build up (a, b) or decline (c, d) of clickable MC (a, c) or AP (b, d).

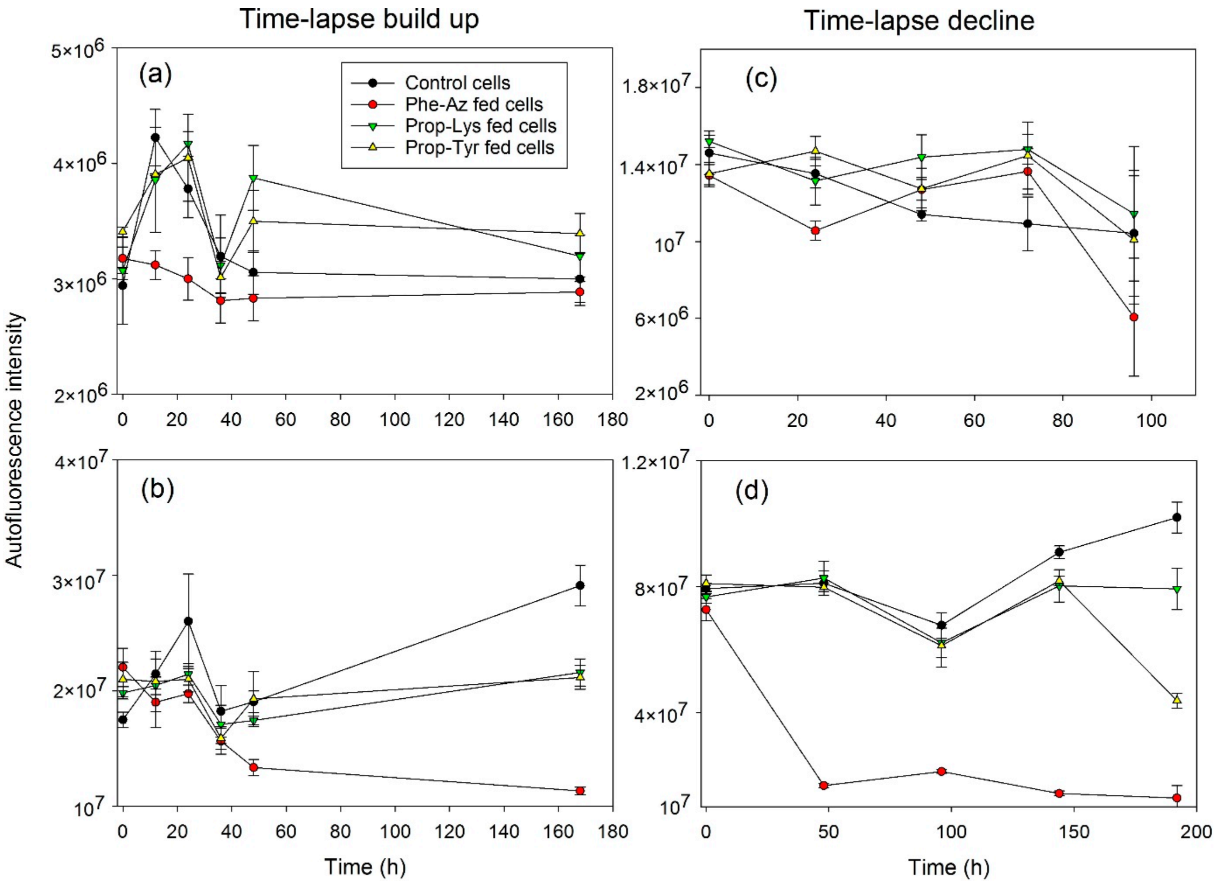

Figure S4: Mean (± SE) autofluorescence intensity of *M. aeruginosa* (a, c) or *P. agardhii* (b, d) per cell during time-lapse experiments related to the build up (a, b) or decline (c, d) of clickable MC (a, c) or AP (b, d).

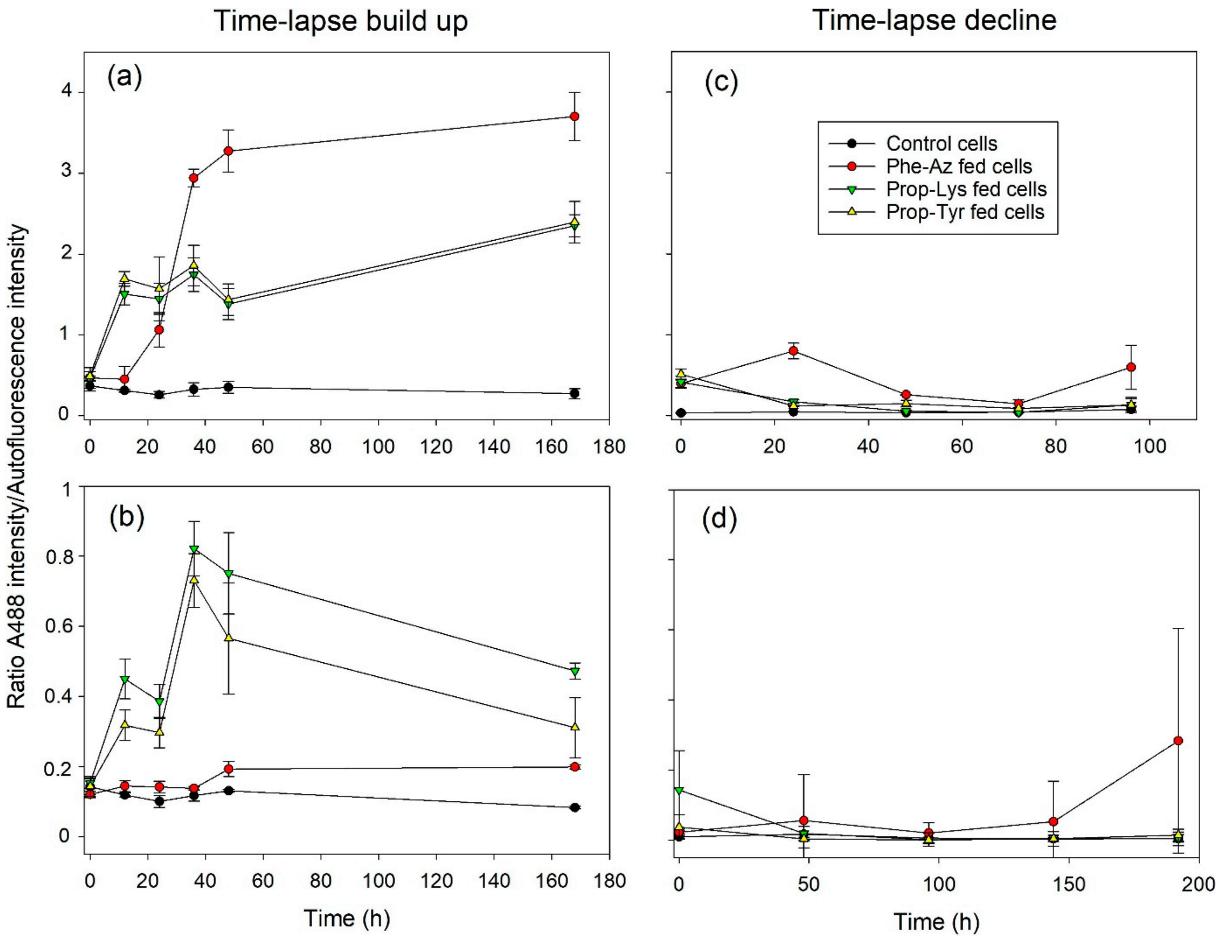

Figure S5: Mean (± SE) ratio of A488 intensity vs. autofluorescence intensity of *M. aeruginosa* (a, c) or *P. agardhii* (b, d) during time-lapse experiments related to the build up (a, b) or decline (c, d) of clickable MC (a, c) or AP (b, d).

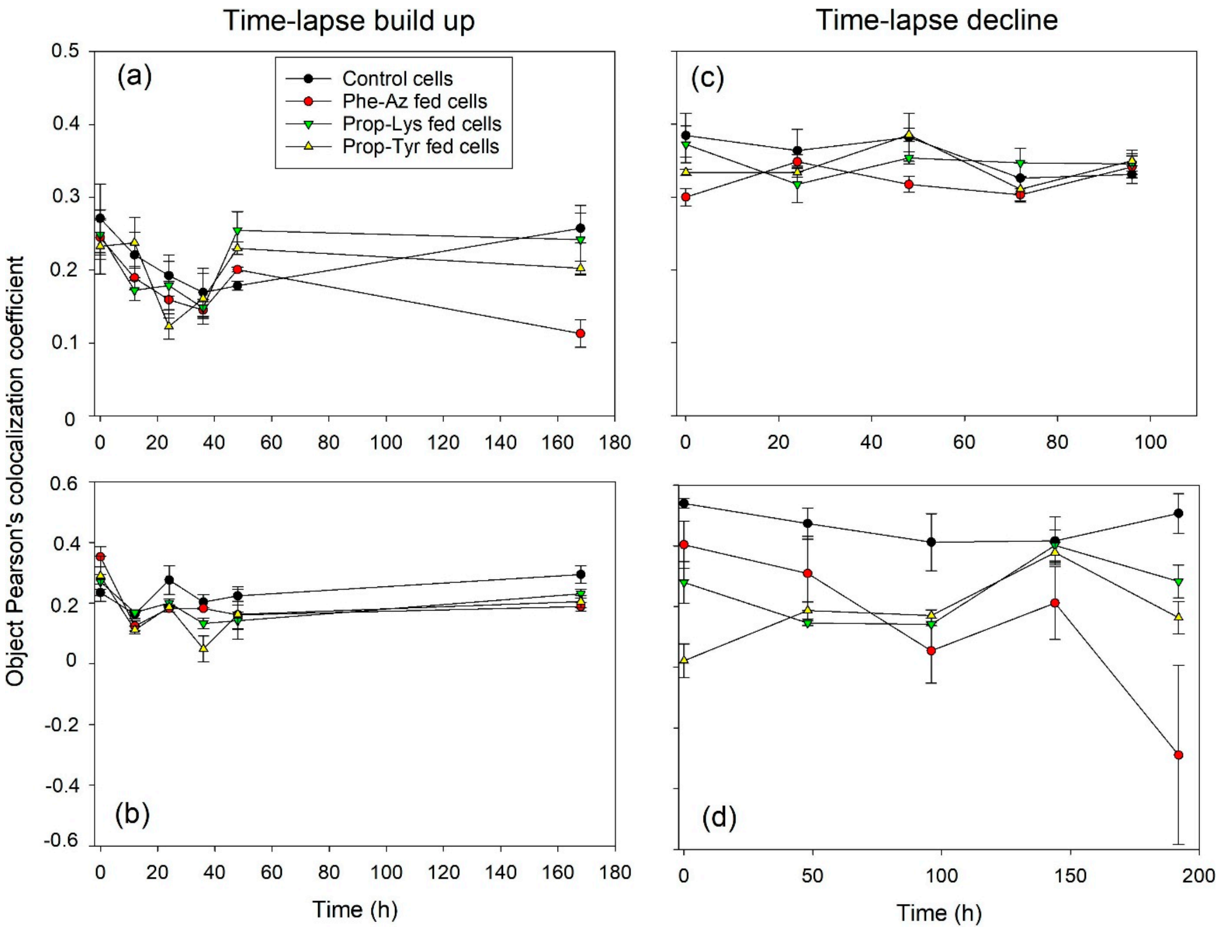

Figure S6: Mean (± SE) of Object Pearson's co-localization coefficient between A488 signal intensity vs. autofluorescence signal intensity of *M. aeruginosa* (a, c) or *P. agardhii* (b, d) during time-lapse experiments related to the build up (a, b) or decline (c, d) of clickable MC (a, c) or AP (b, d).

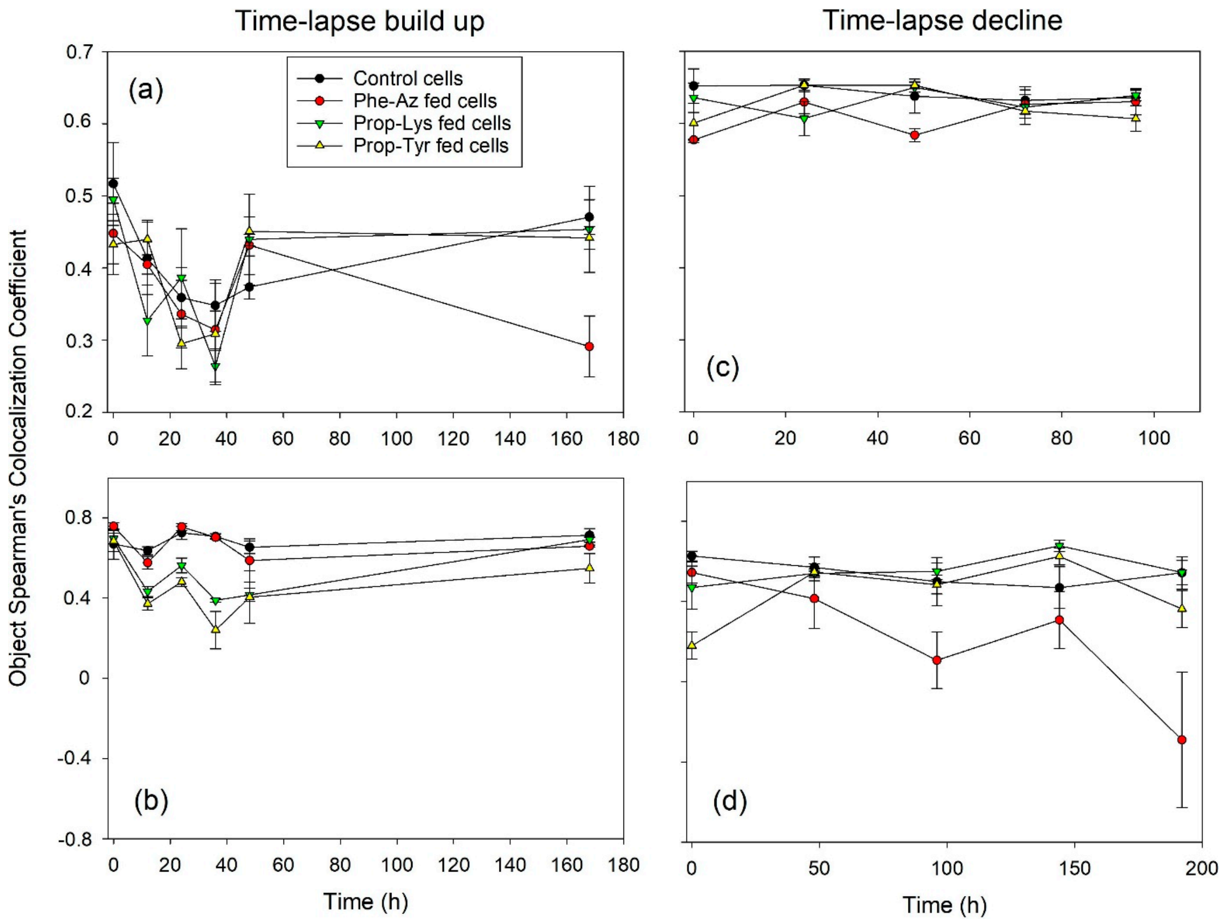

Figure S7: Mean (± SE) of Object Spearman's co-localization coefficient between A488 signal intensity vs. autofluorescence signal intensity of *M. aeruginosa* (a, c) or *P. agardhii* (b, d) during time-lapse experiments related to the build up (a, b) or decline (c, d) of clickable MC (a, c) or AP (b, d).

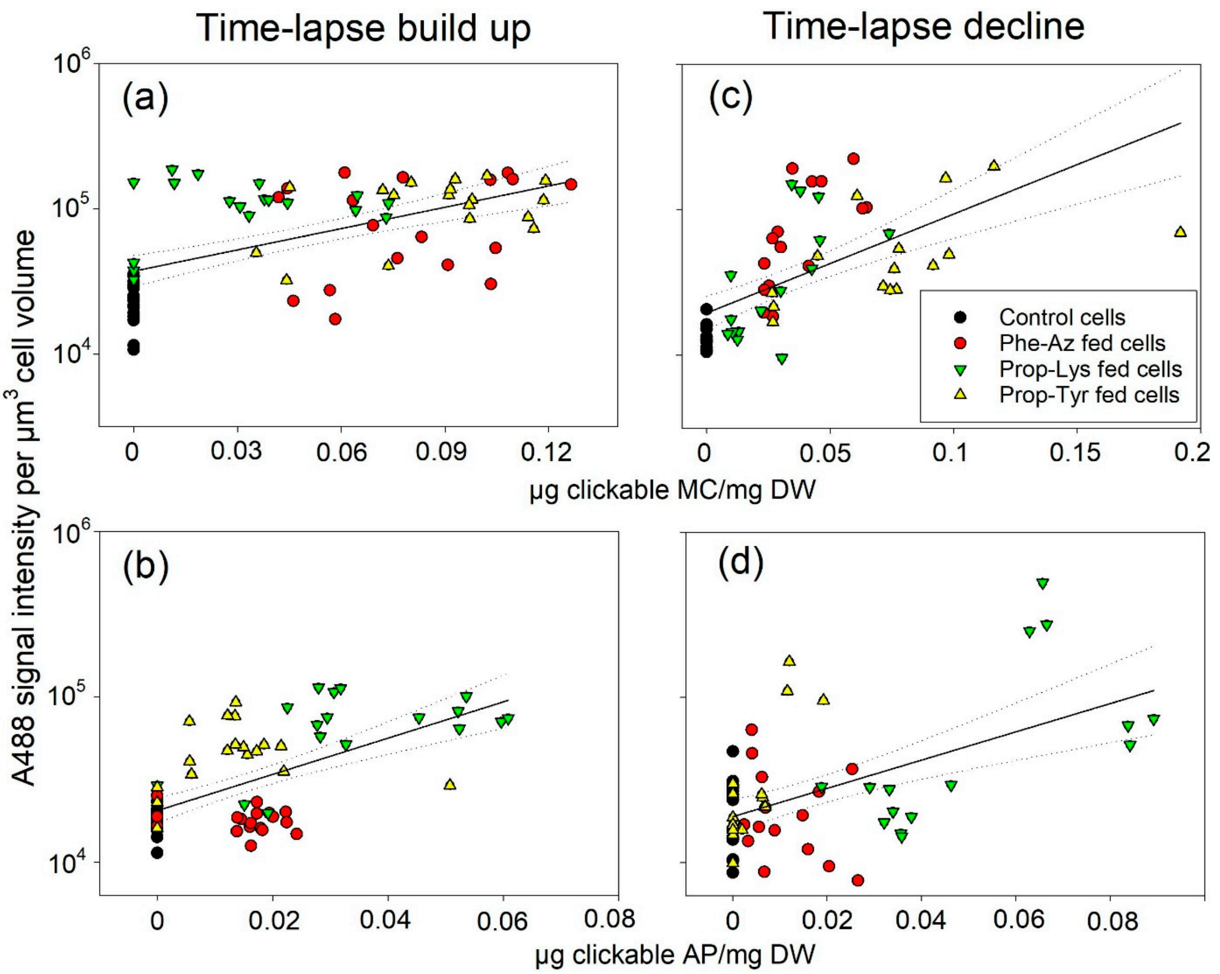

Figure S8: A488 signal intensity per cell volume (in µm³) of *M. aeruginosa* (a, c) or *P. agardhii* (b, d) during time-lapse experiments vs. (a, c) µg of clickable MC or (b, d) µg of clickable AP per mg of dry weight (DW). Details of linear regression lines: y = a + bx, where y is log (x+1) A488 signal intensity and x is log(x+1) of clickable MC/AP per mg of DW as determined by LC-MS previously [23]. Dotted lines indicate 95% confidence intervals. (a) y = 4.57 + 4.89x (R^2 = 0.33, $p < 0.001$), (c) y = 4.29 + 6.81x (R^2 = 0.38, $p < 0.001$), (b) y = 4.31 + 10.97x (R^2 = 0.38, $p < 0.001$), (d) y = 4.28 + 8.55x (R^2 = 0.29, $p < 0.001$).

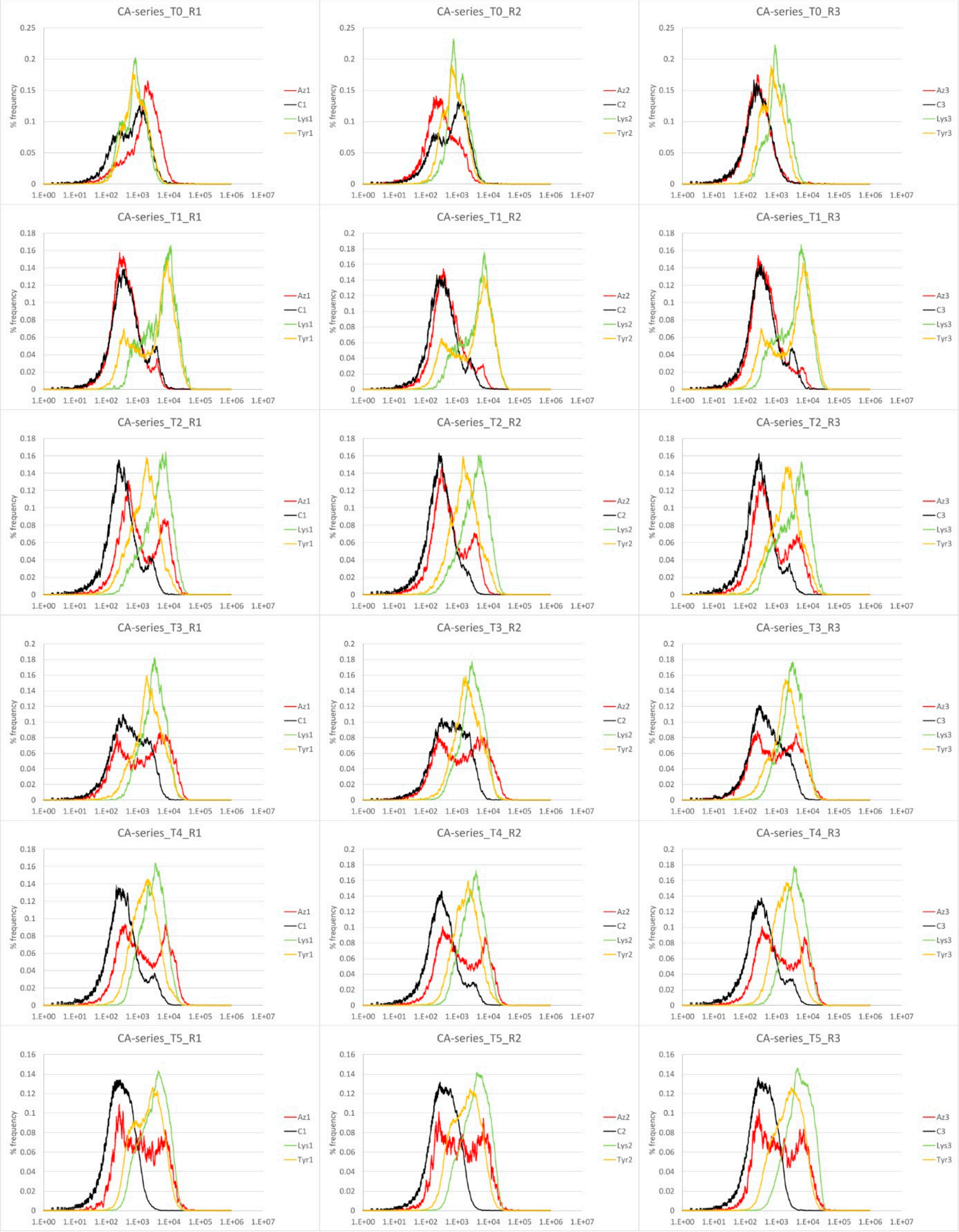

Figure S9: Histograms showing relative frequency (%) of BL1-A fluorescent particles in logarithmic scale (blue laser (BL, 488 nm, 50 mW), (band pass filter at 530 ± 15 nm) as recorded by flow cytometry for *M. aeruginosa* during time-lapse build up experiment in order to quantify labeled subpopulations (three technical replicates are shown).

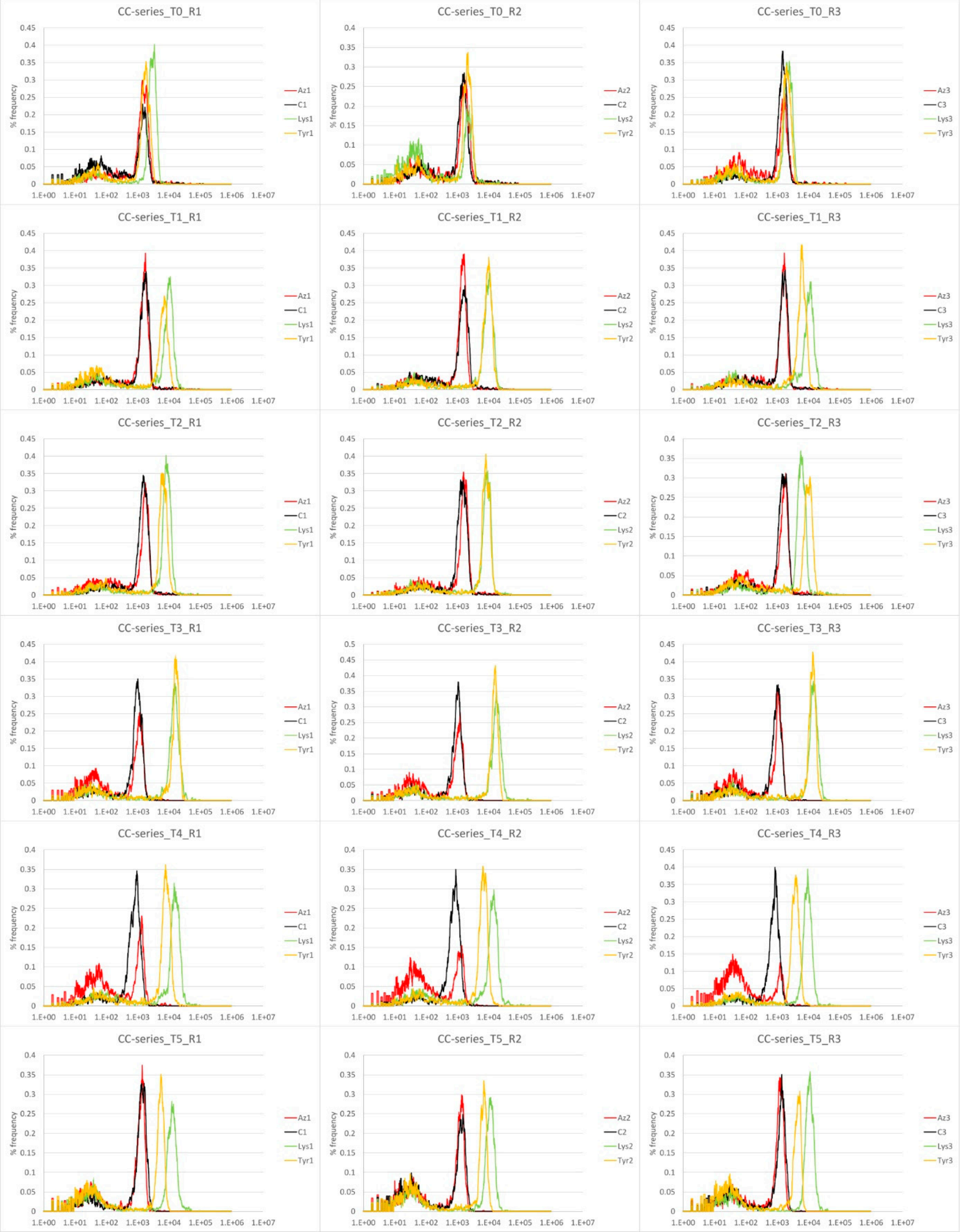

Figure S10: Histograms showing relative frequency (%) of BL1-A fluorescent particles in logarithmic scale (blue laser (BL, 488 nm, 50 mW), (band pass filter at 530 ± 15 nm) as recorded by flow cytometry for *P. agardhii* during time-lapse build up experiment in order to quantify labeled subpopulations (three technical replicates are shown).

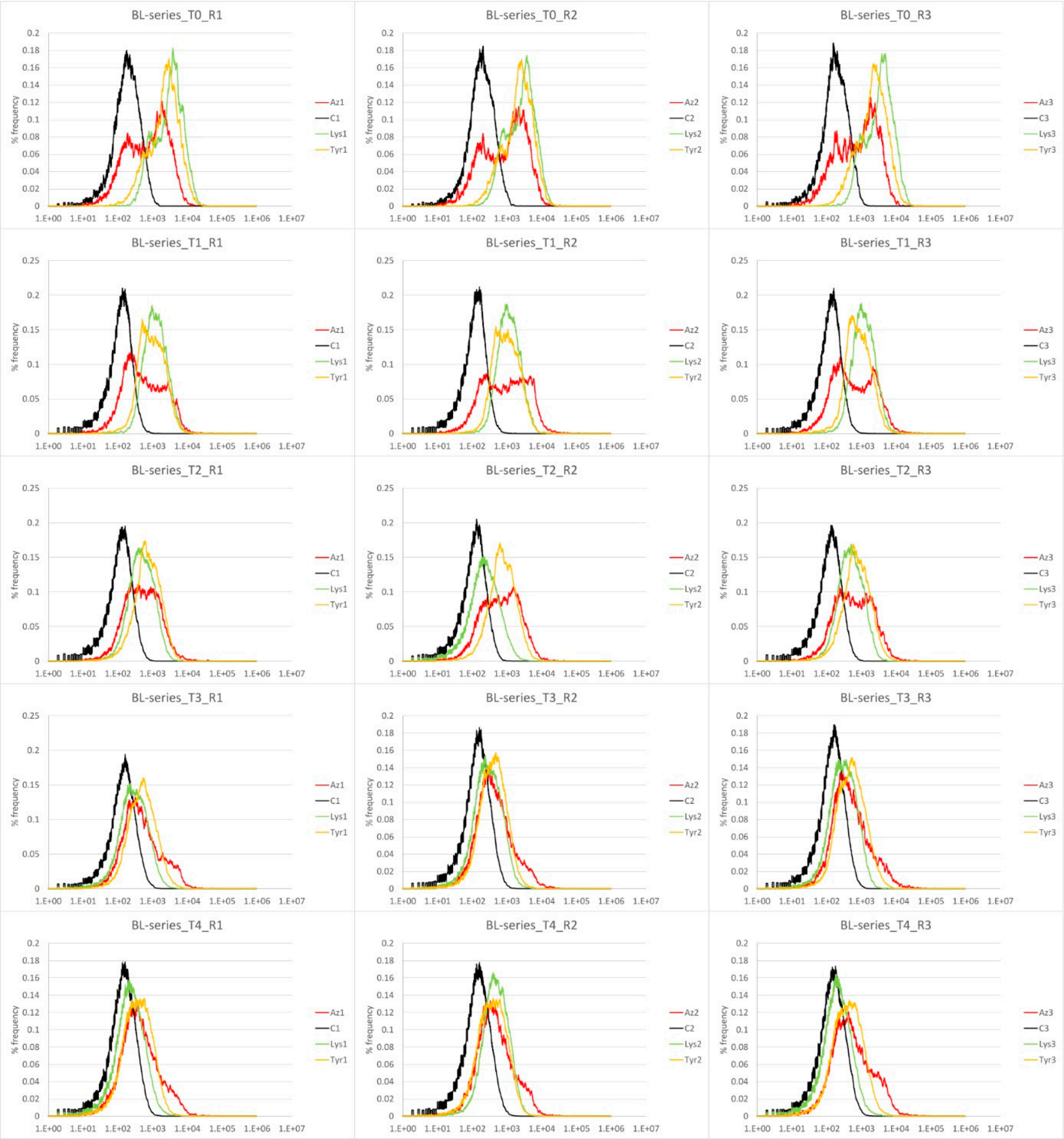

Figure S11: Histograms showing relative frequency (%) of BL1-A fluorescent particles in logarithmic scale (blue laser (BL, 488 nm, 50 mW), (band pass filter at 530 ± 15 nm) as recorded by flow cytometry for *M. aeruginosa* during time-lapse decline experiment in order to quantify labeled subpopulations (three technical replicates are shown).

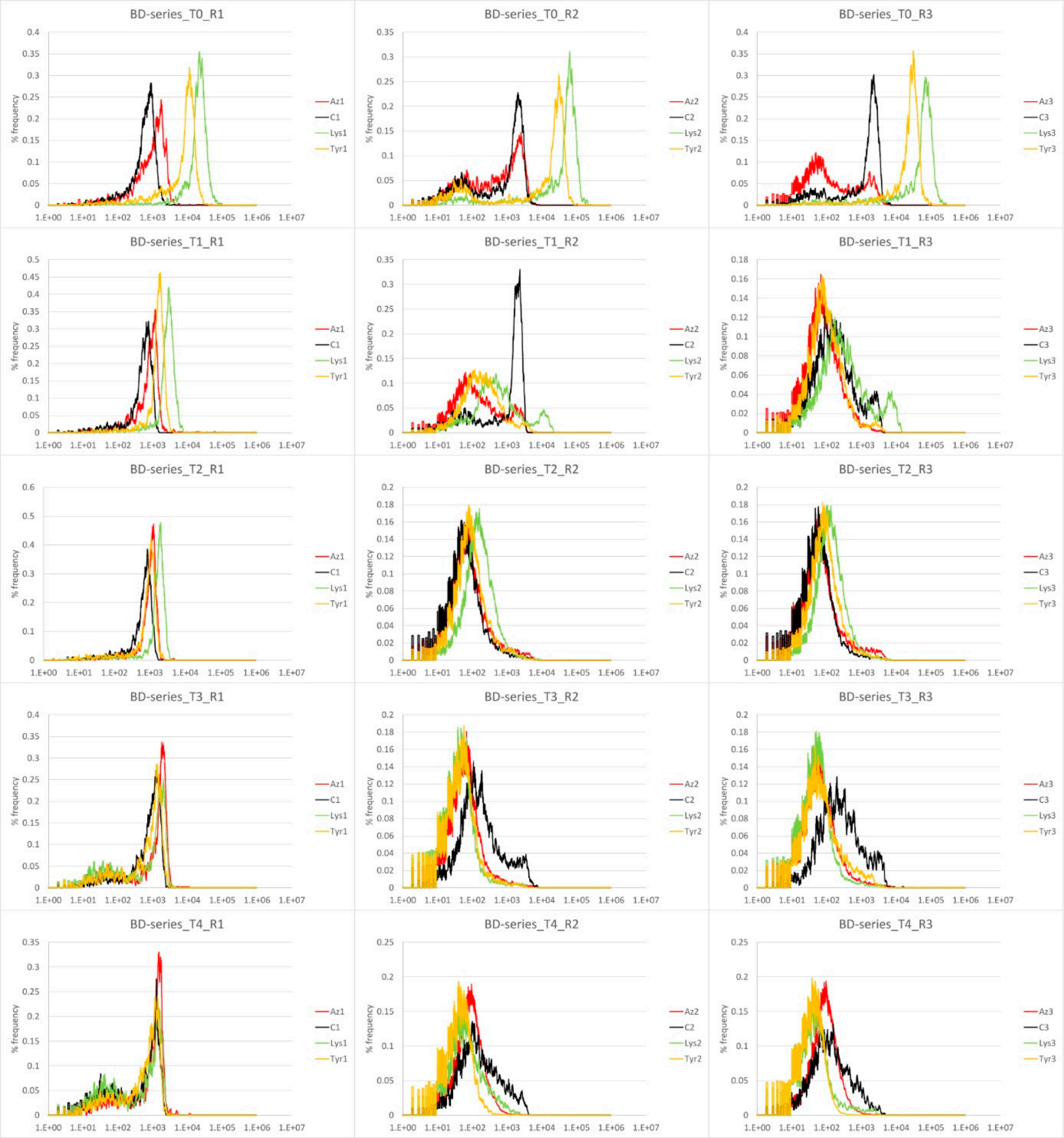

Figure S12: Histograms showing relative frequency (%) of BL1-A fluorescent particles in logarithmic scale (blue laser (BL, 488 nm, 50 mW), (band pass filter at 530 ± 15 nm) as recorded by flow cytometry for *P. agardhii* during time-lapse decline experiment in order to quantify labeled subpopulations (three technical replicates are shown).

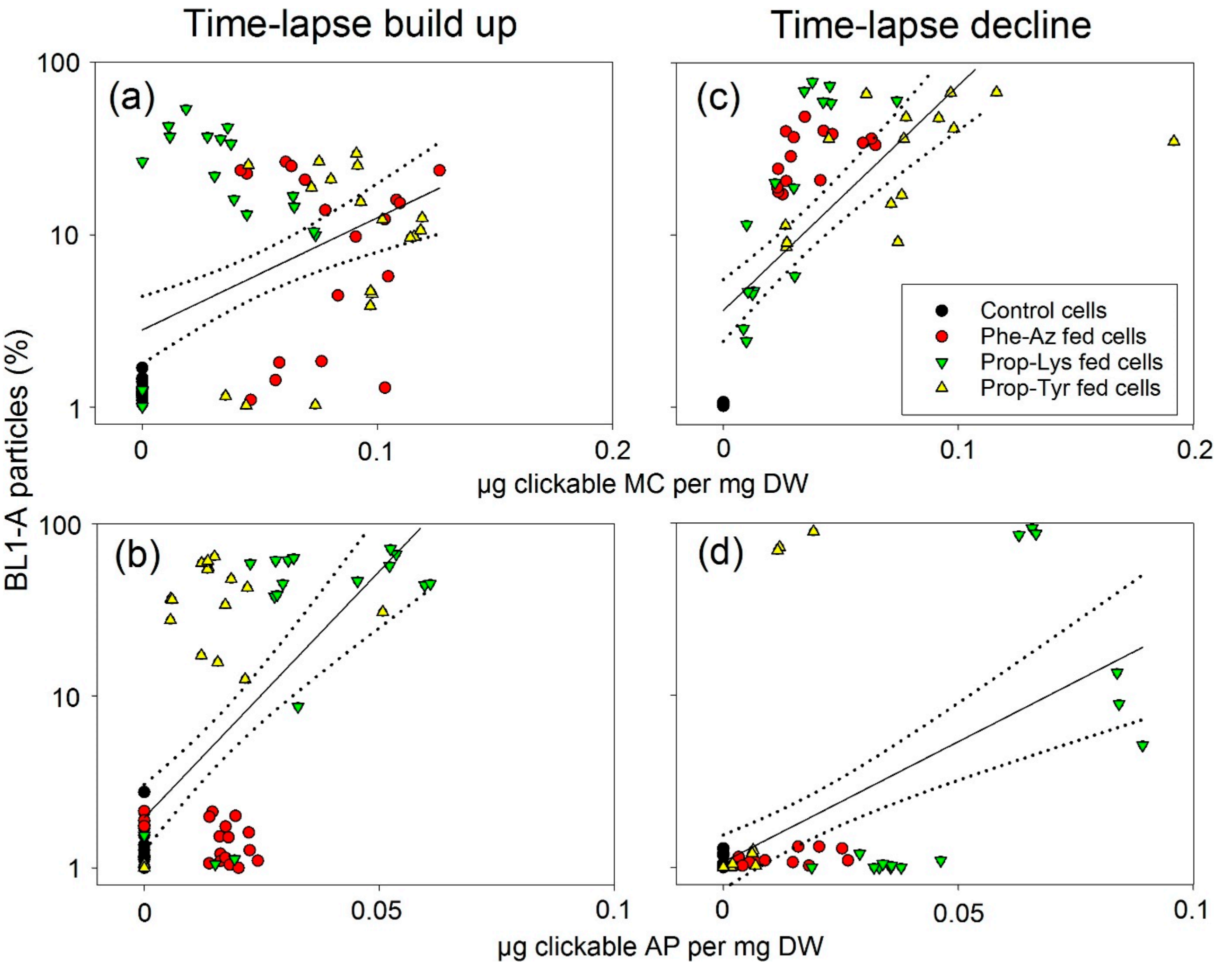

Figure S13: A488 labeled particles (percentage of total particles) of *M. aeruginosa* (a, c) or *P. agardhii* (b, d) during time-lapse experiments vs. percentage of clickable MC (a, c) or AP (b, d). Details of linear regression lines: (a) Y = a + bx, where y is log (x+1) A488 particles and x is log(x+1) µg of clickable MC/AP per mg dry weight (DW) as determined by LC-MS [23]. Dotted lines indicate 95% confidence intervals. Regression curves: (a) y = 0.48 + 6.52x (R^2 = 0.21, $p < 0.001$), (c) y = 0.56 + 13.05x (R^2 = 0.49, $p < 0.001$), (b) y = 0.29 + 28.6x (R^2 = 0.39, $p < 0.001$), (d) y = 0.03 + 14x (R^2 = 0.31, $p < 0.001$), without Phe-Az fed cells (b) y = 0.46 + 30.2x (R^2 = 0.52, $p < 0.001$), (d) y = 0.08 + 13.8x (R^2 = 0.3, $p < 0.001$).

Table S1. Mean ± SE cell diameter and cell volume for each time point as recorded during time-lapse signal build up and decline experiments in cyanobacteria *M. aeruginosa* strain Hofbauer and *P. agardhii* strain no371/1. Two-way RM ANOVA was used to test for differences between treatments: Grouping factor (three non-AA treatments and control), time factor (T0 - T4, T5), three replicates (graphical data are shown in Figure S1, S2).

	M. aeruginosa								***P. agardhii***							
	**Time-lapse build up**				**Time-lapse decline**				**Time-lapse build up**				**Time-lapse decline**			
	**Control**	**Phe-Az**	**Prop-Lys**	**Prop-Tyr**	**Control**	**Phe-Az**	**Prop-Lys**	**Prop-Tyr**	**Control**	**Phe-Az**	**Prop-Lys**	**Prop-Tyr**	**Control**	**Phe-Az**	**Prop-Lys**	**Prop-Tyr**
Cell diameter (μm)																
T0	4.2±0.11	4.2±0.02	4.15±0.06	4.25±0.05	4.00±0.02	4.32±0.12	4.22±0.08	4.20±0.03	3.40±0.03	3.46±0.04	3.46±0.01	3.43±0.02	3.66±0.07	3.67±0.07	3.74±0.1	3.72±0.06
T1	4.42±0.01	4.42±0.02	4.47±0.08	4.41±0.11	4.00±0.05	4.28±0.05	4.19±0.05	4.11±0.1	3.50±0.02	3.50±0.02	3.47±0.02	3.47±0.02	3.60±0.13	3.86±0.05	3.68±0.04	3.62±0.02
T2	4.35±0.07	4.46±0.05	4.51±0.04	4.48±0.05	3.96±0.09	4.52±0.04	4.28±0.07	4.09±0.03	3.53±0.02	3.58±0.02	3.58±0.04	3.52±0.02	3.47±0.2	3.74±0.03	3.47±0.12	3.41±0.11
T3	4.44±0.08	4.56±0.01	4.54±0.08	4.43±0.04	4.09±0.05	4.63±0.03	4.26±0.03	4.19±0.07	3.57±0.02	3.61±0.03	3.60±0.03	3.58±0.06	3.61±0.05	3.73±0.09	3.46±0.04	3.48±0.11
T4	4.25±0.05	4.67±0.03	4.49±0.07	4.52±0.01	4.18±0.06	5.18±0.2	4.19±0.1	4.13±0.05	3.58±0.03	3.67±0.1	3.57±0.02	3.57±0.03	3.56±0.04	3.69±0.08	3.56±0.02	3.23±0.11
T5	4.17±0.09	5.23±0.07	4.37±0.11	4.75±0.07					3.57±0.03	3.56±0.04	3.54±0.04	3.52±0.01				
RM-ANOVA	Grouping factor *p*=0.001; Time factor *p*<0.001; Interaction *p*<0.001; *df*=71				Grouping factor *p*<0.001; Time factor *p*=0.008; Interaction *p*<0.001; *df*=59				Grouping factor *p*=0.084; Time factor *p*<0.001; Interaction *p*=0.965; *df*=71				Grouping factor *p*=0.001; Time factor *p*=0.197; Interaction *p*=0.181; *df*=59			
Cell volume (μm^3)																
T0	39.5±2.9	39.2±0.6	37.9±1.5	40.8±1.4	34.1±0.6	43.1±3.3	40.3±2.3	39.4±1.0	124±3	132±5	128±2	129±1	146±6	145±6	152±9	150±6
T1	45.9±0.3	46.1±0.6	47.8±2.7	45.9±3.6	34.1±1.3	41.8±1.3	39.1±1.4	37.3±2.8	129±3	130±1	130±2	130±1	139±12	164±7	148±4	143±2
T2	43.7±2.1	47.4±1.8	48.9±1.0	47.8±1.7	33.0±2.3	49.0±1.0	41.8±2.2	36.5±1.0	134±1	137±2	138±3	133±3	135±17	157±2	132±11	128±8
T3	46.4±2.6	50.9±0	49.9±3.0	46.2±1.1	36.2±1.2	53.7±0.9	41.2±0.8	38.9±1.9	139±2	143±3	136±2	140±6	143±4	153±8	133±5	131±4
T4	40.6±1.5	54.5±1.1	48.2±2.2	49.2±0.2	39.1±2.0	78.1±9.6	39.3±3.3	37.7±1.4	138±2	143±9	138±3	139±2	139±2	151±5	137±3	114±10
T5	38.3±2.4	76.5±2.9	44.5±3.5	57.2±2.7					137±5	138±4	134±4	133±1				
RM-ANOVA	Grouping factor *p*<0.001; Time factor *p*<0.001; Interaction *p*<0.001; *df*=71				Grouping factor *p*<0.001; Time factor *p*=0.008; Interaction *p*<0.001; *df*=59				Grouping factor *p*=0.083; Time factor *p*=0.01; Interaction *p*=0.98; *df*=71				Grouping factor *p*=0.002; Time factor *p*=0.398; Interaction *p*=0.193; *df*=59			

Table S2. Mean ± SE A488 signal intensity ($\times 10^4$) and autofluorescence ($\times 10^5$) per cell volume (in μm^3) and A488/Autofluorescence ratio for each time point as recorded during time-lapse signal build up and decline experiments in cyanobacteria *M. aeruginosa* and *P. agardhii*. Two-way RM ANOVA was used to test for differences between treatments: Grouping factor (three non-AA treatments and control), time factor (T0 - T4, T5), three replicates (graphical data are shown in Figure 6,7, Figure S5).

	M. aeruginosa								***P. agardhii***							
	**Time-lapse build up**				**Time-lapse decline**				**Time-lapse build up**				**Time-lapse decline**			
	**Control**	**Phe-Az**	**Prop-Lys**	**Prop-Tyr**	**Control**	**Phe-Az**	**Prop-Lys**	**Prop-Tyr**	**Control**	**Phe-Az**	**Prop-Lys**	**Prop-Tyr**	**Control**	**Phe-Az**	**Prop-Lys**	**Prop-Tyr**
A488 signal intensity ($\times 10^4$) per cell volume																
T0	2.7±0.4	3.7±0.8	3.8±0.3	4.1±0.5	1.3±0.1	12.1±1.8	13.6±0.8	16.3±2.1	1.8±0.2	2.0±0.3	2.4±0.3	2.2±0.4	1.8±0.5	1.8±0.9	34.1±7.7	12.3±2.1
T1	2.9±0.3	2.9±0.9	12.3±1.5	12.9±0.6	1.8±0.2	19.1±1.9	5.7±0.9	4.8±0.4	2.0±0.2	2.0±0.1	6.9±0.6	4.8±0.2	2.6±1.1	1.9±0.4	6.5±0.7	2.4±0.1
T2	2.2±0.4	6.1±1.0	11.9±1.7	12.9±2.8	1.2±0.1	6.3±0.4	1.9±0.5	4.9±1.2	1.9±0.1	1.8±0.1	5.9±0.5	4.4±0.5	1.1±0.2	1.3±0.2	2.9±0.03	1.7±0.1
T3	2.1±0.6	16±0.9	10.8±0.6	12.3±2.4	1.3±0.2	3.0±0.7	1.4±0.6	3.2±0.4	1.5±0.2	1.5±0.1	10.2±0.8	8.2±0.5	2.7±0.2	2.4±0.5	1.6±0.1	1.4±0.2
T4	2.6±0.5	16.7±0.5	10.7±1.1	10.2±8.9	1.4±0.1	3.0±0.7	2.2±0.7	2.2±0.3	1.8±0.1	1.8±0.1	9.3±1.5	4.3±0.7	2.8±0.2	4.2±1.4	2.2±0.3	2.4±0.4
T5	2.2±0.5	12.4±0.7	17.0±1.1	14.0±0.5					1.8±0.2	1.6±0.1	7.6±0.3	4.8±1.1				
RM-ANOVA	Grouping factor *p*<0.001; Time factor *p*<0.001; Interaction *p*<0.001; *df*=71				Grouping factor *p*<0.001; Time factor *p*<0.001; Interaction *p*<0.001; *df*=59				Grouping factor *p*<0.001; Time factor *p*=0.002; Interaction *p*<0.001; *df*=71				Grouping factor *p*=0.003; Time factor *p*=0.002; Interaction *p*<0.001; *df*=59			
AF intensity ($\times 10^5$) per cell volume																
T0	0.8±0.0	0.8±0.0	0.8±0.0	0.9±0.0	4.4±0.4	3.2±0.1	3.9±0.2	3.5±0.2	1.4±0.0	1.7±0.0	1.6±0.1	1.6±0.1	5.5±0.1	5.1±0.3	5.1±0.2	5.5±0.1
T1	0.9±0.1	0.7±0.0	0.8±0.1	0.9±0.1	4.0±0.1	2.6±0.2	3.4±0.2	4.1±0.3	1.7±0.1	1.5±0.1	1.6±0.1	1.6±0.2	5.9±0.3	1.0±0.1	5.6±0.4	5.6±0.1
T2	0.9±0.0	0.6±0.0	0.9±0.0	0.9±0.1	3.5±0.1	2.6±0.2	3.5±0.1	3.6±0.1	1.9±0.3	1.5±0.3	1.6±0.1	1.6±0.1	5.2±0.6	1.4±0.0	4.8±0.1	4.8±0.3
T3	0.7±0.1	0.6±0.0	0.6±0.1	0.7±0.0	3.0±0.4	2.6±0.2	3.7±0.2	3.8±0.6	1.3±0.2	1.1±0.2	1.3±0.1	1.1±0.1	6.4±0.3	1.0±0.1	6.1±0.6	6.4±0.3
T4	0.8±0.0	0.5±0.0	0.8±0.1	0.7±0.1	2.8±0.9	1.0±0.6	3.1±1.0	2.7±0.9	1.4±0.1	1.0±0.1	1.3±0.0	1.4±0.2	7.4±0.4	0.8±0.2	5.9±0.6	4.0±0.1
T5	0.8±0.0	0.4±0.0	0.7±0.0	0.6±0.0					2.1±0.1	0.8±0.1	1.6±0.1	1.6±0.1				
RM-ANOVA	Grouping factor *p*<0.001; Time factor *p*=0.001; Interaction *p*<0.017; *df*=71				Grouping factor *p*=0.003; Time factor *p*=0.234; Interaction *p*=0.176; *df*=59				Grouping factor *p*=0.003; Time factor *p*=0.003; Interaction *p*<0.001; *df*=71				Grouping factor *p*<0.001; Time factor *p*=0.011; Interaction *p*<0.001; *df*=59			
A488 intensity/AF intensity ratio																
T0	0.37±0.1	0.47±0.1	0.46±0.0	0.49±0.1	0.03±0.0	0.4±0.1	0.41±0.1	0.51±0.1	0.14±0.0	0.12±0.0	0.15±0.0	0.14±0.0	0.03±0.0	0.04±0.0	0.68±0.1	0.23±0.0
T1	0.31±0.0	0.45±0.2	1.51±0.1	1.69±0.1	0.04±0.0	0.8±0.1	0.17±0.1	0.12±0.1	0.12±0.0	0.14±0.0	0.45±0.1	0.32±0.0	0.04±0.0	0.2±0.1	0.12±0.0	0.04±0.0
T2	0.26±0.0	1.06±0.2	1.45±0.2	1.57±0.4	0.03±0.0	0.26±0.0	0.05±0.0	0.15±0.0	0.1±0.0	0.14±0.0	0.39±0.1	0.3±0.0	0.02±0.0	0.1±0.0	0.06±0.0	0.04±0.0
T3	0.33±0.1	2.94±0.1	1.75±0.2	1.86±0.3	0.04±0.0	0.15±0.1	0.04±0.1	0.09±0.1	0.12±0.0	0.14±0.0	0.82±0.1	0.73±0.1	0.04±0.0	0.26±0.1	0.03±0.0	0.02±0.0
T4	0.35±0.1	3.28±0.3	1.38±0.2	1.44±0.2	0.08±0.0	0.6±0.3	0.13±0.3	0.13±0.3	0.13±0.0	0.19±0.0	0.75±0.1	0.57±0.2	0.04±0.0	0.66±0.3	0.04±0.0	0.06±0.0
T5	0.28±0.1	3.70±0.3	2.35±0.1	2.4±0.3					0.08±0.0	0.2±0.0	0.47±0.0	0.31±0.1				

RM-ANOVA	Grouping factor *p*<0.001; Time factor *p*<0.001; Interaction *p*<0.001; *df*=71	Grouping factor *p*<0.001; Time factor *p*=0.078; Interaction *p*<0.001; *df*=59	Grouping factor *p*<0.001; Time factor *p*<0.001; Interaction *p*<0.001; *df*=71	Grouping factor *p*=0.017; Time factor *p*=0.076; Interaction *p*<0.001; *df*=59

Table S3. Mean ± SE of co-localization coefficients between A488 signal intensity vs. autofluorescence signal intensity for each time point as recorded during time-lapse experiments using pulsed feeding of non-AAs in order to observe the build up or decline of clickable MC or AP. Two-way RM ANOVA was used to test for differences between treatments: Grouping factor (three non-AA treatments and control), time factor (T0 - T4, T5), three replicates (graphical data are shown in Figure S6, S7).

	M. aeruginosa								***P. agardhii***							
	**Time-lapse build up**				**Time-lapse decline**				**Time-lapse build up**				**Time-lapse decline**			
	**Control**	**Phe-Az**	**Prop-Lys**	**Prop-Tyr**	**Control**	**Phe-Az**	**Prop-Lys**	**Prop-Tyr**	**Control**	**Phe-Az**	**Prop-Lys**	**Prop-Tyr**	**Control**	**Phe-Az**	**Prop-Lys**	**Prop-Tyr**
Object Pearson's co-localization coefficient																
T0	0.27±0.1	0.25±0.0	0.25±0.0	0.23±0.0	0.38±0.0	0.3±0.0	0.37±0.0	0.33±0.0	0.23±0.0	0.35±0.0	0.27±0.0	0.29±0.1	0.54±0.0	0.4±0.1	0.28±0.1	0.02±0.1
T1	0.22±0.0	0.19±0.0	0.17±0.0	0.24±0.0	0.36±0.0	0.35±0.0	0.32±0.0	0.33±0.0	0.16±0.0	0.12±0.0	0.17±0.0	0.11±0.0	0.47±0.1	0.31±0.1	0.15±0.0	0.19±0.0
T2	0.19±0.0	0.16±0.0	0.18±0.0	0.12±0.0	0.38±0.0	0.32±0.0	0.35±0.0	0.39±0.0	0.28±0.1	0.18±0.0	0.2±0.0	0.19±0.0	0.41±0.1	0.05±0.1	0.14±0.0	0.17±0.0
T3	0.17±0.0	0.15±0.0	0.15±0.0	0.16±0.0	0.33±0.0	0.3±0.0	0.35±0.0	0.31±0.0	0.2±0.0	0.18±0.0	0.13±0.0	0.05±0.0	0.42±0.1	0.21±0.1	0.4±0.1	0.38±0.0
T4	0.18±0.0	0.2±0.0	0.25±0.0	0.23±0.0	0.33±0.0	0.34±0.0	0.35±0.0	0.35±0.0	0.22±0.0	0.16±0.0	0.14±0.0	0.16±0.1	0.51±0.1	-0.29±0.3	0.28±0.1	0.16±0.1
T5	0.26±0.0	0.11±0.0	0.24±0.1	0.2±0.0					0.29±0.0	0.19±0.0	0.23±0.0	0.21±0.0				
RM-ANOVA	Grouping factor *p*=0.288; Time factor *p*=0.03; Interaction *p*=0.033; *df*=71				Grouping factor *p*=0.105; Time factor *p*=0.006; Interaction *p*=0.216; *df*=59				Grouping factor *p*=0.022; Time factor *p*=0.003; Interaction *p*=0.061; *df*=71				Grouping factor *p*=0.006; Time factor *p*=0.229; Interaction *p*=0.002; *df*=59			
Object Spearman's co-localization coefficient																
T0	0.52±0.1	0.45±0.0	0.5±0.0	0.43±0.0	0.65±0.0	0.58±0.0	0.64±0.0	0.6±0.0	0.67±0.1	0.76±0.1	0.7±0.0	0.69±0.1	0.63±0.0	0.54±0.1	0.47±0.1	0.18±0.07
T1	0.41±0.1	0.4±0.0	0.33±0.1	0.44±0.0	0.65±0.0	0.63±0.0	0.61±0.0	0.65±0.0	0.64±0.0	0.58±0.0	0.43±0.0	0.37±0.0	0.57±0.1	0.41±0.2	0.54±0.0	0.55±0.04
T2	0.36±0.0	0.34±0.1	0.39±0.1	0.3±0.0	0.64±0.0	0.58±0.0	0.65±0.0	0.65±0.0	0.73±0.0	0.76±0.0	0.56±0.0	0.48±0.0	0.5±0.1	0.11±0.1	0.55±0.0	0.48±0.05
T3	0.35±0.0	0.31±0.0	0.26±0.0	0.31±0.1	0.63±0.0	0.63±0.0	0.62±0.0	0.62±0.0	0.71±0.0	0.7±0.0	0.39±0.0	0.24±0.1	0.47±0.1	0.31±0.1	0.68±0.0	0.63±0.04
T4	0.37±0.0	0.43±0.0	0.44±0.1	0.45±0.0	0.64±0.0	0.63±0.0	0.64±0.0	0.61±0.0	0.65±0.0	0.59±0.0	0.42±0.0	0.4±0.1	0.54±0.1	-0.29±0.3	0.54±0.1	0.36±0.09
T5	0.47±0.0	0.29±0.0	0.45±0.1	0.44±0.0					0.71±0.0	0.66±0.0	0.69±0.0	0.55±0.1				
RM-ANOVA	Grouping factor *p*=0.727; Time factor *p*=0.006; Interaction *p*<0.033; *df*=71				Grouping factor *p*=0.035; Time factor *p*=0.532; Interaction *p*=0.183; *df*=59				Grouping factor *p*=0.001; Time factor *p*<0.001; Interaction *p*=0.011; *df*=71				Grouping factor *p*=0.013; Time factor *p*=0.25; Interaction *p*<0.001; *df*=59			

Table S4. Mean ± SE proportion of clickable MC in total MC (sum of clickable and natural MC variants) or of clickable AP in total AP (sum of clickable and natural AP variants) for each time point as recorded during time-lapse experiments using pulsed feeding of non-AAs in order to observe the build up or decline of clickable MC or AP. Two-way RM ANOVA was used to test for differences between treatments: Grouping factor (three non-AA treatments and control), time factor (T0 - T4, T5), three replicates (graphical data are shown in Kurmayer and Moron [23], Figure 1, 2).

	M. aeruginosa								***P. agardhii***							
	**Time-lapse build up**				**Time-lapse decline**				**Time-lapse build up**				**Time-lapse decline**			
	**Control**	**Phe-Az**	**Prop-Lys**	**Prop-Tyr**	**Control**	**Phe-Az**	**Prop-Lys**	**Prop-Tyr**	**Control**	**Phe-Az**	**Prop-Lys**	**Prop-Tyr**	**Control**	**Phe-Az**	**Prop-Lys**	**Prop-Tyr**
Percentage of clickable MC									Percentage of clickable AP							
T0	0±0	2.4±0.2	0±0	3.6±0.1	0±0	16.0±0.3	2.1±0.2	43.2±1.5	0±0	0±0	3.1±1.7	0±0	0±0	17.6±1.2	58.2±1.4	4.3±0.3
T1	0±0	12.6±1.5	1.5±0.6	20.3±1.1	0±0	14.1±0.5	1.6±0.1	35.7±0.6	0±0	9.4±2.0	36.9±1.0	2.3±0.5	0±0	11.2±0.8	67.8±0.5	1.5±0.1
T2	0±0	12.2±0.9	2.4±0.1	33.2±2.4	0±0	9.6±0.1	2.4±0.1	22.0±0.4	0±0	10.3±1.3	61.6±0.8	8.7±1.2	0±0	3.3±1.2	49.7±1.8	1.0±0.5
T3	0±0	15.1±0.5	3.0±0.4	48.3±0.5	0±0	8.6±0.7	0.9±0.1	15.6±0.4	0±0	17.3±0.8	56.3±2.2	3.6±0.6	0±0	2.9±0.3	53.9±1.8	0±0
T4	0±0	21.3±1.4	3.5±0.2	60.8±1.5	0±0	6.6±0.4	0.6±0.1	6.6±0.1	0±0	16.9±1.3	50.8±13	6.3±0.1	0±0	1.7±0.3	47.3±4.3	0±0
T5	0±0	21.2±3.0	3.5±0.2	89.5±2.0					0±0	21.1±1.2	84.2±0.6	5.6±0.4				
RM-ANOVA	Grouping factor p=0.001; Time factor p<0.001; Interaction p<0.001; df=71				Grouping factor p=0.001; Time factor p<0.001; Interaction p<0.001; df=59				Grouping factor p=0.001; Time factor p<0.001; Interaction p<0.001; df=69				Grouping factor p=0.001; Time factor p<0.001; Interaction p<0.001; df=59			
Clickable MC in percentage of control									Clickable AP in percentage of control							
T0	0±0	5.2±1.2	0±0	4.0±0.9	0±0	9.2±2.5	2.3±0.3	33.3±8.4	0±0	0±0	2.6±1.3	0±0	0±0	11.7±1.1	45.3±0.9	4.1±0.8
T1	0±0	9.8±1.1	0.8±0.3	23.6±4.8	0±0	8.6±1.1	3.4±0.3	42.7±2.6	0±0	3.5±0.5	20.5±2.3	1.6±0.5	0±0	7.2±0.6	58.1±1.4	1.2±0.1
T2	0±0	11.1±0.1	2.7±0.2	39.4±2.5	0±0	4.0±0.1	3.2±0.5	48.4±23	0±0	6.6±1.4	47.3±3.4	7.7±1.6	0±0	3.8±2.0	71.1±19	0.8±0.4
T3	0±0	12.9±2.8	3.9±0.2	49.8±3.2	0±0	1.3±0.3	0.8±0.1	29.5±0.8	0±0	9.2±0.9	32.9±3.7	2.6±0.4	0±0	3.5±0.5	59.8±1.3	0±0
T4	0±0	11.3±2.0	3.5±0.3	31.5±1.5	0±0	1.2±0.1	0.8±0	7.8±0.1	0±0	11.2±1.7	44.1±11	5.8±0.4	0±0	1.6±0.3	43.5±1.4	0±0
T5	0±0	1.7±0.7	2.8±0.2	52.2±5.1					0±0	21.3±0.7	85.2±4.5	4.1±0.2				
RM-ANOVA	Grouping factor p=0.001; Time factor p<0.001; Interaction p<0.001; df=71				Grouping factor p=0.001; Time factor p=0.057; Interaction p=0.075; df=59				Grouping factor p=0.001; Time factor p<0.001; Interaction p<0.001; df=69				Grouping factor p<0.001; Time factor p=0.316; Interaction p=0.059; df=59			

Table S5. Mean ± SE proportion of clickable MC in µg per mg of DW or of clickable AP in µg per mg of DW for each time point as recorded during time-lapse experiments using pulsed feeding of non-AAs in order to observe the build up or decline of clickable MC or AP. Two-way RM ANOVA was used to test for differences between treatments: Grouping factor (three non-AA treatments and control), time factor (T0 - T4, T5), three replicates (graphical data are shown in Kurmayer and Moron [23], Suppl. Figure S5, S6).

	M. aeruginosa								***P. agardhii***							
	**Time-lapse build up**				**Time-lapse decline**				**Time-lapse build up**				**Time-lapse decline**			
	**Control**	**Phe-Az**	**Prop-Lys**	**Prop-Tyr**	**Control**	**Phe-Az**	**Prop-Lys**	**Prop-Tyr**	**Control**	**Phe-Az**	**Prop-Lys**	**Prop-Tyr**	**Control**	**Phe-Az**	**Prop-Lys**	**Prop-Tyr**
clickable MC in µg per mg of DW									clickable AP in µg per mg of DW							
T0	0±0	0.23±0.04	0±0	0.13±0.03	0±0	0.14±0.02	0.10±0.01	0.24±0.05	0±0	0±0	0.03±0.01	0±0	0±0	0.06±0	0.16±0	0.03±0.01
T1	0±0	0.15±0.02	0.05±0.02	0.18±0.04	0±0	0.11±0.02	0.13±0.03	0.23±0.02	0±0	0.04±0	0.09±0.02	0.04±0	0±0	0.04±0	0.22±0.01	0.02±0
T2	0±0	0.21±0.02	0.09±0	0.29±0.02	0±0	0.07±0	0.07±0.01	0.29±0.14	0±0	0.04±0	0.07±0	0.05±0	0±0	0.01±0	0.08±0.02	0±0
T3	0±0	0.25±0.06	0.12±0.02	0.29±0.01	0±0	0.08±0.01	0.03±0	0.19±0	0±0	0.04±0	0.06±0	0.03±0	0±0	0.01±0	0.09±0	0±0
T4	0±0	0.26±0.03	0.18±0.01	0.25±0	0±0	0.06±0	0.02±0	0.06±0	0±0	0.05±0	0.10±0.02	0.03±0	0±0	0.01±0	0.1±0.02	0±0
T5	0±0	0.12±0.02	0.03±0.01	0.21±0.02					0±0	0.05±0	0.14±0.01	0.01±0				
RM-ANOVA	Grouping factor $p<0.001$; Time factor $p<0.001$; Interaction $p<0.001$; df=71				Grouping factor $p<0.001$; Time factor p=0.013; Interaction p=0.143; df=59				Grouping factor $p<0.001$; Time factor $p<0.001$; Interaction $p<0.001$; df=69				Grouping factor $p<0.001$; Time factor $p<0.001$; Interaction $p<0.001$; df=59			
clickable MC in µg per mg of DW (percentage of control)									clickable AP in µg per mg of DW (percentage of control)							
T0	0±0	27.6±5.3	0±0	15.3±3.7	0±0	21.7±2.9	14.7±1.3	36.5±7.1	0±0	0±0	11.8±6	0±0	0±0	14.0±1.1	39.8±0.7	8.2±1.5
T1	0±0	35.0±5.4	10.9±5.5	41.6±8.5	0±0	23.8±3.8	27.7±5.5	47.5±3.5	0±0	12.1±1	27.1±4.9	10.9±1.3	0±0	9.3±0.6	52.6±1.2	3.6±0.1
T2	0±0	43.2±3.7	18.1±0.7	60.2±5.1	0±0	13.6±0.5	13.1±1.3	57.4±27.5	0±0	18.3±2.7	31.5±1.7	21.3±1.6	0±0	8.2±2.6	64.8±17.1	2.4±1.2
T3	0±0	50.0±10.8	23.8±3.9	58.0±2.9	0±0	14.4±2.5	5.4±0.3	35.8±0.7	0±0	13.2±0.7	23.4±2.1	11.1±0.4	0±0	9.3±0.6	56.1±1.2	0±0
T4	0±0	35.5±4.1	24.5±1.1	34.9±0.1	0±0	10.8±0.1	4.3±0.2	12.5±0.1	0±0	14.6±1.3	37.0±4.7	10.8±0.4	0±0	6.6±1.9	29.9±11.5	0±0
T5	0±0	30.7±4.4	8.1±1.4	51.8±4.0					0±0	25.7±2.2	74.1±3.7	6.9±0.1				
RM-ANOVA	Grouping factor $p<0.001$; Time factor $p<0.001$; Interaction $p<0.001$; df=71				Grouping factor $p<0.001$; Time factor p=0.016; Interaction p=0.141; df=59				Grouping factor $p<0.001$; Time factor $p<0.001$; Interaction $p<0.001$; df=69				Grouping factor $p<0.001$; Time factor p=0.291; Interaction p=0.035; df=59			

Table S6. Mean ± SE percentage (%) of BL1-A fluorescent particles and cell (particle) counts (blue laser (BL, 488 nm, 50 mW), (band pass filter at 530 ± 15 nm) as recorded by flow cytometry during time-lapse experiments using pulsed feeding of non-AAs in order to observe the build up or decline of clickable MC or AP. Two-way RM ANOVA was used to test for differences between treatments: Grouping factor (three non-AA treatments and control), time factor (T0 - T4, T5), three replicates).

	M. aeruginosa								***P. agardhii***							
	**Time-lapse build up**				**Time-lapse decline**				**Time-lapse build up**				**Time-lapse decline**			
	**Control**	**Phe-Az**	**Prop-Lys**	**Prop-Tyr**	**Control**	**Phe-Az**	**Prop-Lys**	**Prop-Tyr**	**Control**	**Phe-Az**	**Prop-Lys**	**Prop-Tyr**	**Control**	**Phe-Az**	**Prop-Lys**	**Prop-Tyr**
Percentage of BL1-A fluorescent particles																
T0	0.5±0.1	1.8±1.5	0.2±0.08	0.07±0.04	0.02±0	35.6±2	72±2.6	65.6±0.4	1.0±0.4	0.9±0.1	0.2±0.2	0.02±0.02	0.2±0.1	0.2±0.1	87±2.6	76±6
T1	0.3±0.1	0.6±0.2	27.5±4.5	26.2±1.3	0.05±0.1	39.4±4.2	58.3±0.5	44.8±2.1	0.9±0.1	1.0±0.1	45.5±1.0	25.8±10	0.01±0	0.1±0.1	8.2±2.5	0.2±0.1
T2	0.3±0.1	10.7±4.8	36.2±2.5	11.6±1.7	0.03±0	34.1±3.4	13.9±4.6	34.7±0.4	0.5±0.2	0.7±0.1	27.3±9.8	28.5±8.9	0.02±0.01	0.1±0.03	0.1±0.1	0.03±0.02
T3	0.2±0.01	20±4.3	14.3±1.1	9.8±0.8	0.04±0	18.5±1.1	3.7±0.05	12.8±2.4	0.2±0	0.1±0.04	59.3±0.8	55.2±1.4	0.1±0.06	0.1±0.2	0.01±0.01	0±0
T4	0.2±0.04	14±0.6	10.7±1.5	3.4±0.3	0.06±0	19.3±2	4.6±3	8.6±0.9	0.1±0.04	0.31±0.1	65.9±2.4	50.8±11	0±0	0.05±0.03	0.01±0.01	0±0
T5	0.1±0.03	22.8±0.7	43.5±4.9	20.6±1.9					0±0	0.05±0.03	47.5±4.2	32.3±2.9				
RM-ANOVA	Grouping factor $p<0.001$; Time factor $p<0.001$; Interaction $p<0.001$; $df=71$				Grouping factor $p<0.001$; Time factor $p<0.001$; Interaction $p<0.001$; $df=59$				Grouping factor $p<0.001$; Time factor $p<0.001$; Interaction $p<0.001$; $df=69$				Grouping factor $p<0.001$; Time factor $p<0.001$; Interaction $p<0.001$; $df=59$			
Number of BL1-A particles (cells)																
T0	76±20	254±206	23±11	10±7	5±1	3165±418	13326±415	11967±808	20±6	16±5	4±2	0.3±0.3	4±2	6±1	1839±26	1679±93
T1	56±11	107±44	2873±545	5333±175	19±4	6532±880	9850±707	9542±540	22±2	21±3	1055±11	591±205	0.3±0.2	2.8±1.3	359±162	6.4±3.5
T2	55±17	1561±662	5487±745	2001±99	16±1	5304±558	6079±1022	15371±414	11±4	16±4	622±224	707±238	2.4±1.8	8.1±4.4	12.9±6.7	2.9±1.7
T3	38±7	1120±186	1650±278	1524±81	27±6	3291±286	2084±221	8748±2816	3±0.1	3±1	1165±3	1086±21	3.7±1.9	10.3±3.8	1.2±1.2	0±0
T4	75±12	2482±276	3013±600	1210±115	46±9	3695±715	2226±913	5827±968	2±1	8±2	1547±11	1107±226	0±0	3.9±1.8	1±1	0.7±0.7
T5	99±31	1381±28	48563±8127	20351±3220					0.1±0.1	1±0.6	1318±62	935±78				
RM-ANOVA	Grouping factor $p<0.001$; Time factor $p<0.001$; Interaction $p<0.001$; $df=71$				Grouping factor $p<0.001$; Time factor $p<0.001$; Interaction $p<0.001$; $df=59$				Grouping factor $p<0.001$; Time factor $p<0.001$; Interaction $p<0.001$; $df=69$				Grouping factor $p<0.001$; Time factor $p<0.001$; Interaction $p<0.001$; $df=59$			

References

23. Kurmayer, R.; Morón Asensio, R. Real-time observation of clickable cyanotoxin synthesis in bloom-forming cyanobacteria *Microcystis aeruginosa* and *Planktothrix agardhii*. *Toxins* **2024**, *16*, 526.
